# Mixing Meds and Milk: Evaluation of a Performance Gap Intervention for Provider Education in Breastfeeding and Maternal Medication Use

**DOI:** 10.3390/ijerph20196850

**Published:** 2023-09-28

**Authors:** Kaci Bohn, Alejandra Fernandez, Stephanie Stroever, Dara O’Neil, Joan Enderle, Kaytlin Krutsch

**Affiliations:** 1 School of Pharmacy, Texas Tech University Health Sciences Center, Amarillo, TX 79106, USA; 2 School of Medicine, Texas Tech University Health Sciences Center, Lubbock, TX 79430, USA; 3 American Heart Association, Dallas, TX 75231, USAjoan.enderle@heart.org (J.E.); 4 School of Medicine, Texas Tech University Health Sciences Center, Amarillo, TX 79106, USA

**Keywords:** breastfeeding, lactation, pharmacology, medication, education

## Abstract

The need for maternal medications is a known barrier to breastfeeding. Though most medications are compatible with lactation, healthcare providers use abundant caution, often viewing medications and breastfeeding as mutually exclusive. A dual intervention of an educational webinar and access to a mobile app for lactation pharmacology was used to enhance provider familiarity, confidence, and access to knowledge in medication use during breastfeeding. Surveys were administered before, one week after, and three months after the webinar to evaluate performance gap improvement. Usage data of the mobile app was collected over twelve months to monitor topic engagement. Results suggested the interventions temporarily increased provider confidence in maternal medication use during lactation; however, the increase was not sustained at three months. Even with one-time training and lactation-specific mobile app access, simply providing an informational resource is insufficient to support evidence-informed care for lactating patients. Longitudinal training on evidence-based medication safety is critical to care for the lactating dyad.

## 1. Introduction

Most major health organizations recommend breastfeeding for two years in response to known maternal and infant health outcomes for the breastfeeding dyad. For the mother, breastfeeding reduces the incidence of breast and ovarian cancer, type 2 diabetes mellitus, hyperlipidemia, stroke, and more [1,2]. For the infant, benefits include a reduction in asthma/wheezing, obesity, type 2 diabetes, and increased immunological response [3]. Considering the numerous health benefits, one could assume breastfeeding rates would be high in the USA. However, the CDC reports that in 2019, 83% of mothers initiated breastfeeding, with rates dropping to 55% and 35% at six months and one-year respectively [4,5]. Interventions aimed at increasing breastfeeding duration and intensity have become the focus of many programs across the country and internationally [6,7]. In addition, the use of galactagogue herbal products is increasing as more women desire to breastfeed [8]. Unfortunately, a prominent reason for the cessation of breastfeeding is the risk of medication exposure to the infant through breast milk [1]. While realizing infant risk is influenced by multiple factors, such as genetics and environmental factors [9], many healthcare providers advise mothers to either discontinue breastfeeding or stop medication treatments, both of which negatively impact the health of the mother [10,11]. One study highlighted evidence-based breastfeeding support as a primary influence on a woman’s success with long-term breastfeeding [12]. The benefit to the mother’s health, the risk to the infant, and the opportunity cost of breastfeeding must be considered by the practitioner when prescribing medications. However, only a limited number of medications are genuinely incompatible with breastfeeding [8].

Since the 1980s, recommendations have been to allow short-term use of medications during breastfeeding if drug levels in the breast milk are sufficiently low. If not possible, mothers were, and still are, told to either stop the drug or discontinue breastfeeding [13]. When attempting to determine reasons for breastfeeding cessation, Kirkland et al. determined that maternal medication use was second only to the partner’s desire for the mother to stop [14]. In the USA, data is limited regarding prescription use in lactating mothers. Most studies are either too small in sample size [15] or are unsuccessful in differentiating between pregnant and breastfeeding women [16].

The Centers for Disease Control (CDC) supported a national program to reduce racial and ethnic health disparities in the county of El Paso, Texas, through a Racial and Ethnic Approaches to Community Health (REACH) grant awarded to the American Heart Association (AHA). As a part of the REACH grant’s nutrition strategy, AHA sought to better prepare healthcare providers in El Paso county on how to support breastfeeding mothers and infants. Through participatory action research, several focus areas were determined. In three focus groups, medication use was perceived as a major barrier to successful lactation and contributed to discontinuing breastfeeding in the El Paso community (data thus far unpublished). Other areas of focus of the REACH initiative included pediatric resident training in culturally sensitive communication to support breastfeeding, developing a more substantial foundational medical knowledge of breastfeeding benefits, and increased support of breastfeeding while managing risks from maternal medication for both breastfeeding moms and their babies.

Since medication use is a substantial obstacle to breastfeeding throughout the first year, the aim of this project was to partner with key opinion leaders in lactation pharmacology at the InfantRisk Center at Texas Tech University Health Sciences Center in Amarillo (IRC) to enhance provider familiarity, confidence, and access to knowledge in medication use during lactation [17]. The authors’ aim is to assess the success of the AHA REACH educational intervention for improving providers’ familiarity and confidence in providing recommendations for safe medication use for breastfeeding patients.

## 2. Materials and Methods

### 2.1. Program Evaluation Design

This is a pre/post-program evaluation of the translational REACH project, which consisted of two interventions: (1) an educational webinar and (2) twelve months of access to the lactation-specific medication reference mobile app, InfantRisk HCP (Health Care Provider, Texas Tech University Health Sciences Center, Lubbock, TX, USA). The Texas Tech University Health Sciences Center (TTUHSC) Institutional Review Board (IRB) referred the ethical review of this project to the TTUHSC Quality Improvement Review Board (QIRB). QIRB approved this project on 21 July 2021 (QI-21059).

The educational event was hosted by the AHA and performed by content experts in lactation pharmacology. A live in-person lecture was planned but was moved online due to the COVID-19 pandemic. With this format, the webinar was opened to the public, but only providers in El Paso were followed for the performance gap intervention. Data were collected through three surveys and metrics gathered through the Apple Store platform. Survey development was guided by Bloom’s taxonomy as a framework to evaluate three core areas: familiarity, confidence, and utilization [18]. Surveys were conducted the week before the educational webinar, one week after the webinar, and three months post-webinar. To preemptively mitigate response bias, the survey was designed to be straightforward and non-leading, ensure confidentiality, and offer participants email reminders to increase completion rates.

Invitations to attend the “Mixing Meds + Milk” program were extended to community providers, but participation in the program was voluntary. The only inclusion criterion was to be a provider of care to patients in El Paso County. Training contents are subject to copyright and a strict no-sharing policy. Learning objectives included understanding the importance of breastfeeding for maternal and infant health, identifying types of medications transmitted in breastmilk, assessing safe medications for use in lactation, and describing situations where infants are at greater risk of harm from maternal medications. Informational fliers with the training description, date, time, and registration information were distributed to health systems in El Paso, TTUHSC, the Binational Breastfeeding Coalition, and the local lactation and pediatrics communities.

### 2.2. Educational Intervention

Two training events were held, the pilot event in August 2021 and a second in June 2022. Both events were webinars explaining current literature regarding safe medication use, common disease states, and drug classes often used in breastfeeding. The 2021 pilot webinar was a live lecture by Dr. Thomas Hale, a key opinion leader in lactation pharmacology, over one hour. In this webinar, participants were given an access code for InfantRisk HCP (a lactation pharmacology mobile application) after the event; therefore, they did not have access to download or use the app until after the event. The 2022 event was adjusted based on participant feedback from the pilot session. Registrants were given access to the app before the webinar after completing the pre-event survey. The webinar was split into two sections: Dr. Hale focused on the same lecture material in a prerecorded session, and Dr. Krutsch appended his lecture with a live in-app demonstration to improve participant download and navigation.

### 2.3. Survey Data

Pre- and post-intervention data were collected using structured online surveys. Post-intervention data were collected one week (Post-Survey 1) and three months (Post-Survey 2) after the educational webinar. The primary intention of Post-Survey 1 was to fulfil requirements for continuing education and AHA webinar standards, which were not included in this program evaluation; however, items related to confidence were opportunistically surveyed at this time point. Pre- and Post 2 surveys included questions about the provider’s familiarity and confidence in making medication decisions within their breastfeeding patient population. The surveys primarily consisted of Likert-scale items augmented by multiple-choice and open-ended questions.

Survey questions were asked regarding the familiarity and confidence of providers with evidence-supported medication safety in breastfeeding women in common conditions, including conditions such as mastitis, UTI, depression, and postpartum pain. The same question was asked for chronic conditions, including conditions such as hypertension, diabetes, chronic pain, and lastly unique conditions, including conditions such as transplant and STEMI. Only a subset of survey items specific to our aim are reported in this manuscript; a full list of survey items and schedule is available. See Supplementary Materials.

Each response was assigned a rank, with 1 indicating the lowest level of agreement and 4 representing the highest level of agreement. The ranked data were subsequently used for analysis. Extent of breastfeeding experience was coded as large amount of experience (numerical value 4), moderate amount of experience (value 3), small amount of experience (value 2), and no experience (value 1). Frequency of medication encounters in breastfeeding women was also converted to numerical values, such as several times per week (coded value 4), once per week (value 3), 2–3 times per month (value 2), and monthly (value 1). In addition, familiarity and confidence responses were coded as extremely (value 4), very (value 3), slightly (value 2), and not at all (value 1). When determining how likely providers were to integrate training information into their future practice, responses were considered as very likely (value 4), somewhat likely (value 3), somewhat unlikely (value 2), and very unlikely (value 1). Participants were also asked to rate the presenter and training quality as very good (value 4), good (value 3), fair (value 2), and poor (value 1).

Participants were included in the analytic set if they answered the pre-survey and one other survey (either Post-Survey 1, Post-Survey 2, or both). After accounting for these conditions, 48 participants remained in the total analytic data set. Listwise deletion for missing data further reduced the sample size for each item. The sample ranged from 11 participants (complete cases for familiarity with common medications) to 39 (complete cases for confidence with common medications). We performed post-hoc power calculations using G*Power (Heinrich-Heine-Universität Düsseldorf, Düsseldorf, Germany) to estimate achieved power to detect a moderate effect (d = 0.5) with a one-tailed hypothesis, an alpha error probability of 0.05, and the parent distribution classified as min ARE. For items with 11 complete cases, we achieved only 41.0% power to detect a moderate difference between pre-survey and post-survey scores. However, items with 39 complete cases achieved 88.4% power. The analytic sample size for each item is included in Table 1

Analyses were completed in Stata/MP version 17.0 (StataCorps, LLC., College Station, TX, USA) with listwise deletion for missing data. We tested differences in non-parametric ordinal data using the Wilcoxon signed-rank test to account for repeated measures between the pre-program survey and post-program survey 1, as well as pre-program survey and post-program survey 2. We interpreted the exact *p*-value due to small sample size (n < 200) with alpha = 0.05.

### 2.4. App Data

App usage data are collected from users who have agreed to share their diagnostics and usage information with app developers through Google App Analytics. The Google Analytics platform does not track user-specific profiles, only data sufficient to help developers understand how their users engage with their specific app. To protect user privacy, app analytics only shows data after a certain number of data points are available and cannot be used to track people across apps or websites.

All app users agreed to the terms and conditions of usage, which included data collection upon download. All other information collected was used in the standard monitoring of the app, met standards of data privacy by the app platforms, and was disclosed to users. App users could also opt out of data sharing within their operating system on their mobile devices. With any electronic data collection, there is a risk of loss of data confidentiality. To mitigate this risk, participants were given a code to the app, which created their identities as participants in the program. Only data from Apple app users were generated with enough volume to maintain anonymity to aggregate data; therefore, data from Android devices will not be reported.

## 3. Results

### 3.1. Survey Data

#### 3.1.1. Sample Features

**Figure 1:**
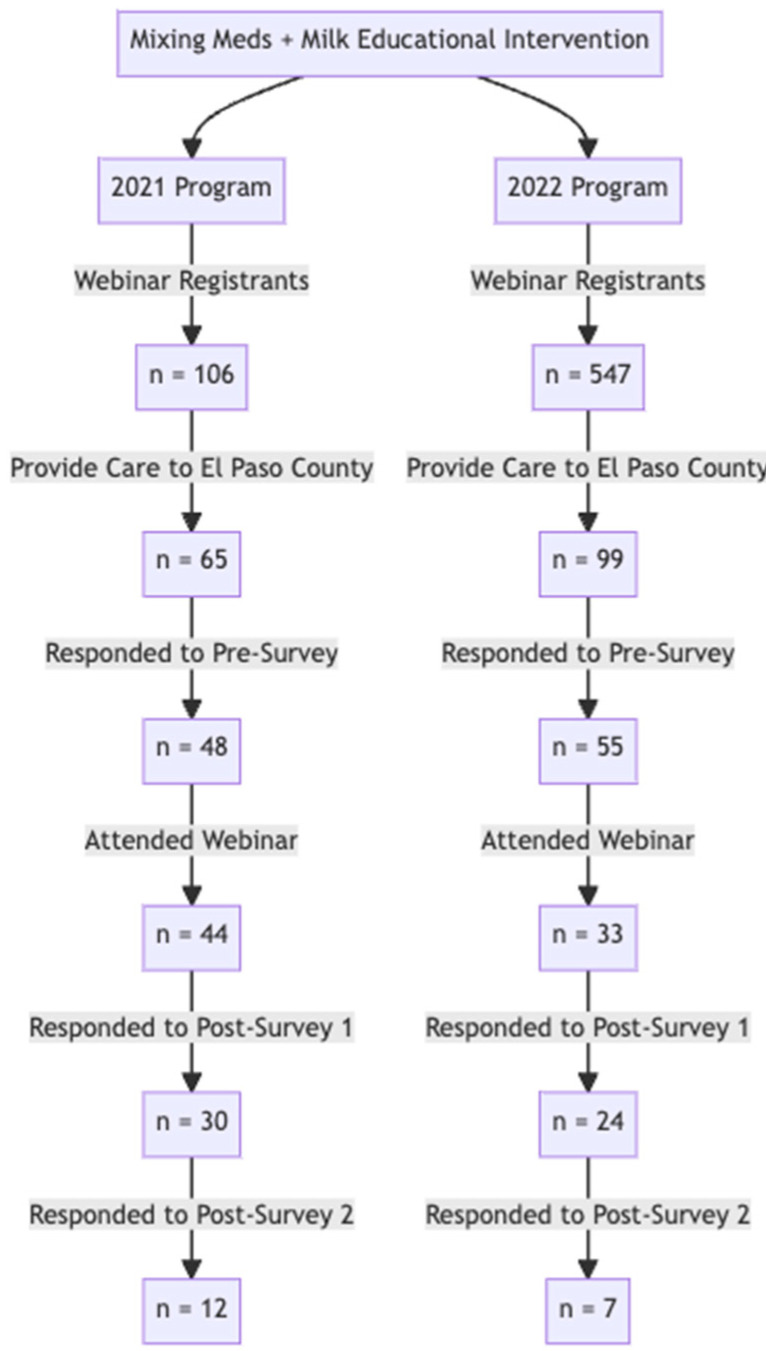
The flow diagram describes the engagement metrics of webinars held in 2021 and 2022. In 2021, there were 65 registrants who provided care to El Paso patients. Of these, 48 responded to the pre-survey. The engagement with survey assessments significantly decreased at the three-month post program evaluation to 12 respondents (Post-Survey 2). In 2022, a similar pattern was observed. Of 55 providers in El Paso county who completed the pre-survey, only 7 completed Post-Survey 2.

The complete flow of participants throughout the program and assessment for both years can be found in Figure 1. The 2021 survey group contained 48 responses to the pre-survey, 30 responses to Post-Survey 1, and 12 responses to Post-Survey 2 (for a total of n = 90). Similarly, the 2022 number of survey responses also declined from the pre-survey (55) to Post-Survey 1 (24) and Post-Survey 2 (7).

The proportion of characteristics exhibited by the baseline and analytic samples were compared using the chi-square test; no characteristics demonstrated a significant difference. See Table 2.

#### 3.1.2. Sample Familiarity and Confidence

**Figure 2:**
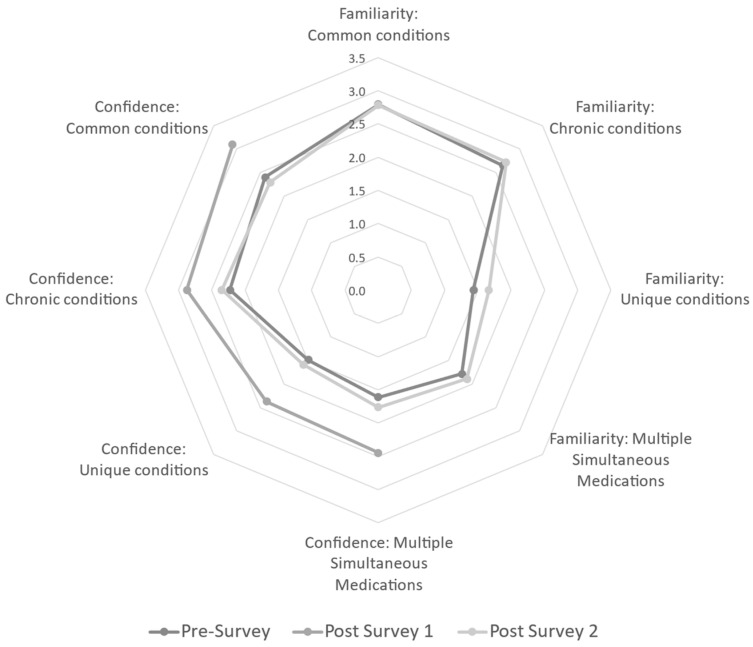
Radar graph of the mean responses to Likert survey items assessing familiarity and confidence for each survey. Post-Survey 1 did not assess familiarity, only items related to confidence were available at all three points. Confidence significantly increased for all related items from baseline to Post-Survey 1 (1 week following the webinar); however, these gains were lost by Post-Survey 2 (3 months following the webinar). Gains were most noticeable for more complicated scenarios, such as unique conditions and multiple simultaneous medications.

To answer the specific question of whether an instructional intervention can increase participant familiarity in medication safety in multiple conditions/situations, baseline values were assessed via the pre-survey. At baseline (pre-survey), responding participants were equally confident when recommending medication to breastfeeding women for common conditions, chronic conditions, unique conditions, and multiple simultaneous medications. A majority of participants in the analytic sample at baseline (total n = 48) reported being either “slightly familiar” or “very familiar” with common conditions (62.5%) and chronic conditions (77.1%). However, there is a shift toward less familiarity with the majority responding either “slightly familiar” or “not at all familiar” with unique conditions (91.7%) and in situations of multiple medication usage (87.6%). Data suggest participants are more comfortable with commonly used/prescribed medications compared with those not as common. Familiarity was not assessed again until Post-Survey 2; Post-Survey 1 was primarily administrative. For Post-Survey 2, 30 participants did not answer familiarity questions. Though not statistically significant using listwise deletion for missing data, there was a shift to the majority of responses being “slightly familiar” or “very familiar” with common (27.1%) and chronic conditions (33.4%). Regarding familiarity of unique conditions and multiple medication usage, participants repeated a non-significant decrease in familiarity responding either “no familiarity” or “slightly familiar” (32.2% and 31.3%, respectively). Though the sample size is small, this analytic sample demonstrated that there was not a sustained increase in familiarity following the program intervention. See Figure 2.

To assess participant confidence in making medication recommendations, the pre-survey determined the majority of providers in the analytic sample were either “slightly confident” or “very confident” in both common conditions (79.1%) and chronic conditions (72.9%). The same pattern of decreased confidence in unique conditions and multiple simultaneous medications was seen, with the majority responding either “slightly confident” or “not at all confident” (81.3% and 83.4%, respectively). The authors were curious to see if the post-survey showed a transient and/or sustained increase in confidence post-intervention, so confidence items were included in the administrative Post-Survey 1 without overly burdening respondents with additional research items. Both Post-Survey 1 and Post-Survey 2 data decreased in sample size compared to the pre-survey (n = 41 and n = 17, respectively). At one week post-intervention (Post-Survey 1), there was a significant increase in confidence for all sub-questions (common, chronic, or unique conditions, and multiple simultaneous medications). Unfortunately, analysis assessing the persistence of confidence from baseline to three months reflected no statistical difference. See Figure 2.

Participants were asked at pre-survey and at three months how often certain resources were used when addressing the issue of medication use in breastfeeding women. There was no significant reported change in how often the respondent used each type of reference by three months after the intervention. The authors report the consistent use of lactation-specific references as the most selected option in all surveys. If comparing the percentage of responses to using this type of reference, 85.4% and 83.3% responded that this was the most used type of reference. The lack of change in utilization to a lactation-specific reference was disappointing and suggested that participants did not adjust or feel a need to alter what they had been doing before the intervention and training. The failure to change resource utilization could explain the lack of sustained confidence in lactation pharmacology and would also suggest further investigation and strengthening of peer-reviewed medication in lactation studies.

#### 3.1.3. Responses to Training

When asked how likely the attendee would be to integrate training material into his/her practice (n = 54), 87% responded very likely, 9% responded somewhat likely, and only 4% responded unlikely. This suggests that the training was meaningful and/or beneficial to 96% of participants. Attendees were then asked if the webinar (n = 19) and/or the InfantRisk HCP app (n = 14) were helpful. The groups responded that 95% found the webinar helpful and 93% found the app helpful. It is encouraging that such a high percentage found the tools used in our intervention beneficial for their practice. Lastly, we assessed how important it is for healthcare providers to have training on medications and breastfeeding (n = 19). 94% of attendees responded that this is very important, 6% responded that this training is important, and zero responded that this material is unimportant. When asked if they believed they needed additional education on lactation pharmacology, 90% responded affirmatively. Free text responses of suggested topics for future education included scenarios including premature infants, infants in neonatal intensive care, and hospitalized infants.

### 3.2. InfantRisk HCP App Data

**Figure 3:**
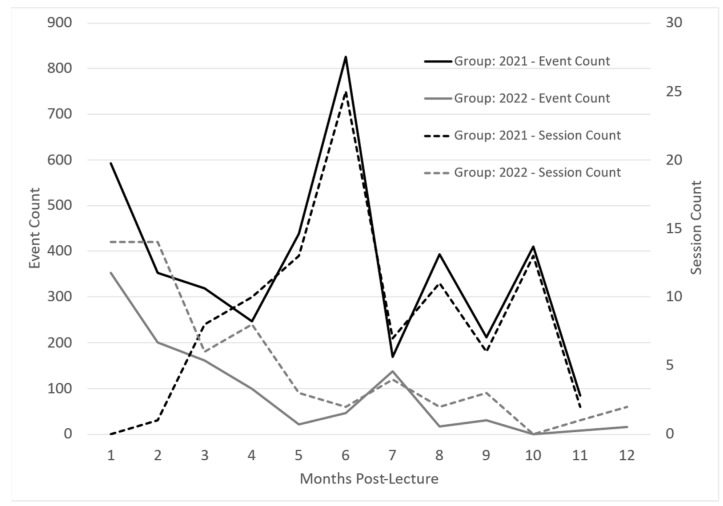
InfantRisk HCP app usage over one year following an interventional lactation pharmacology webinar. Apple defines a session as the number of times the app has been used for at least two seconds. Events are meaningful interactions with the app during a session, such as a button click or page view.

InfantRisk app usage was monitored via Google Analytics for one year to determine any usage patterns from participants. Apple defines app usage in sessions and events. Sessions are the number of times the app was used for at least two seconds. Events are defined as meaningful interactions within the app, such as a button click or page view during a session. Both groups illustrated decreased usage from initial login to three months post-intervention. See Figure 3. This could be due to higher initial interaction when learning to navigate the app and diminishing interest in app contents, which begins to drop as participants return to busy careers and after the completion of Post-Survey 1.

Interestingly, around months 4 to 5, both groups demonstrated increased app usage. One theory suggests that completing the second post-survey reminded participants of the app’s usefulness if they had forgotten it over time. These usage surges also corresponded with the beginning of the cough and cold season, when breastfeeding mothers commonly face medication questions.

Regarding app access for participants, the 2021 cohort was given access to the app after attending the webinar, while the 2022 cohort had access to the app before the webinar to enable participation in the webinar app demonstration. The access of the 2022 group to use the app during the webinar accounts for the higher number of initial sessions, due to participants looking around in the app during the training. The 2022 group, however, had a lower initial number of events, suggesting that participants in 2021 explored the app more as they navigated through the app on their own. The 2021 group’s higher participant number can also partially explain 2021’s higher event count.

## 4. Discussion

### 4.1. Survey Data

Since this project aimed to enhance provider familiarity, confidence, and access to knowledge in medication use during breastfeeding, investigators designed the surveys to assess each of these areas before and after the intervention webinar.

The total number of participantss in attendance at the two webinars was 77; however, not all responded to survey questions, which reduced the sample size. Sample numbers were further decreased to allow for analysis of full responses between the three surveys. Pre-survey sample size was n = 48, which decreased to n = 31 for Post-Survey 1 and n = 17 for Post-Survey 2. The authors acknowledge that small sample sizes led to a lack of significance in the data analyzed as they were not powered to identify even moderate effects for many items. However, promising results indicated a significant and encouraging increase in confidence in the week after the educational intervention. Increased confidence immediately post-webinar could suggest participants either confirmed what they already knew or gained more knowledge/confidence due to the training and/or app use.

Familiarity was only assessed before the intervention and three months after; there was no significant increase in familiarity at these points. These results echo findings from confidence in the pre- and three-months-post surveys. However, as familiarity was not assessed simultaneously with confidence in the first post-survey, any transient increases in familiarity were not observed.

This study also hoped to identify the more common resources used to answer providers’ questions regarding medication use in lactation. Data illustrated that when addressing the issue of medication use in breastfeeding women, respondents preferred utilizing lactation-specific medical references, such as LactMed or InfantRisk HCP, then medical literature, then a trusted colleague, followed by general medical references such as LexiComp. It is encouraging to note that providers with a demonstrated interest in breastfeeding prefer lactation-specific resources to obtain focused and up-to-date information for safe medication use in breastfeeding, thus confirming a critical role for lactation pharmacology resources. The investigators hoped that, minimally, the webinars made abundantly clear the need for lactation-specific resources and up-to-date training for all providers in contact with breastfeeding patients.

As this program assessment suggests that a single educational intervention for lactation pharmacology does not support a sustained increase in provider familiarity and confidence in treating breastfeeding mothers, the authors would hope a more robust intervention could be repeated with a larger sample size to assess effectiveness.

### 4.2. App Data

The innovative addition of the InfantRisk HCP mobile app to the performance gap intervention has provided valuable insights into provider education regarding breastfeeding and maternal medication use. Several critical discussion points have emerged from observing user behavior on the app for the 2021 and 2022 intervention groups.

Both groups illustrated decreased usage from initial login to three months post-intervention. See Figure 3. This could be due to higher initial interaction when learning to navigate the app and diminishing interest in the app contents, which begins to drop as participants return to busy careers and after the completion of Post-Survey 1. The 2021 group participated in the webinar without a hands-on app demonstration. These app users recorded only a handful of sessions, yet there was a high event count. This may indicate a deep exploration by a few particularly invested users. The number of sessions trended upward over the first six months, peaking during December and January, coincidentally marking the peak season for the InfantRisk Call Center.

Conversely, the 2022 group, which had the advantage of an app demonstration during the lecture, seemed less inclined towards exploratory behavior. These users accessed the app with a distinct intent, met their immediate informational needs, and subsequently exited. Their usage (sessions) steadily declined over time, albeit with a modest increase in the winter months (months five through seven). These patterns emphasize the critical nature of timing when it comes to educational interventions. Ideally, providers could be introduced to the app just before the busy season, ensuring they are familiar with its functionalities and ensuring the training is prominent in their minds when the peak period arrives.

Interest and engagement appeared to diminish and stabilize in the second half of the year following the webinar. This aligns with the three-month survey findings, which underscore the potential necessity of introducing reminders or refresher sessions to sustain user engagement.

### 4.3. Limitations

One of the greatest limitations of this study was loss to follow-up and the resulting missingness across items, which dramatically reduced the sample size. Less than half the participants that registered and attended the webinar completed a post-program survey one week later. An even smaller percentage completed the one-month follow-up. The loss to follow up and large degree of missingness (62.5% in Post-Survey 2) make it difficult to draw conclusions about the impact of the program. The power to detect a moderate difference was less than 60% in items with fewer than 18 participants. We can feel confident, however, in the interpretation of the program effect in items with 31 or more participants (80% power).

Response bias was a notable limitation of the study, as participants who completed the later surveys may have been more invested in completing the survey as they recognized the need for additional education on this topic. This bias prevents widespread generalization of the results; however, the accompaniment of app data allows for more confidence in data capture. Other limitations were small sample sizes for both the 2021 (n = 48 pre-survey, n = 30 Post-Survey 1, and n = 12 Post-Survey 2) and 2022 cohorts (n = 55 pre-survey, n = 24 Post-Survey 1, and n = 7 Post-Survey 2) which decreased between each survey point. Due to this decrease, longitudinal data was difficult to analyze.

One challenge we observed in using app data related to data capture. The observed capture rate was less than ideal, making it challenging to determine whether this was due to limited actual app utilization or a higher rate of participant opt-out. Engagement may also represent a disproportionate sample, as a few highly engaged users could be responsible for most data; we cannot see granular app user data. Lastly, individual user data patterns would be beneficial in determining the types of information needed based on provider background, but these data are not accessible yet, creating a limitation of this study.

The COVID-19 pandemic introduced a myriad of challenges and changes in providing healthcare and related education, which may have influenced the educational intervention and assessment. The educational interventions were originally intended as in-person seminars; the virtual delivery mode replaced traditional face-to-face interactions. This shift can impact the assimilation of knowledge and the efficacy of the intervention. Additionally, the heightened stress and altered work dynamics due to the pandemic could influence providers’ responses, potentially affecting their self-reported confidence and familiarity. Moreover, the pandemic might have changed the frequency of medication inquiries, with more mothers potentially seeking advice due to concerns related to COVID-19 and infant safety, which in turn could skew the perception of providers regarding their confidence and familiarity.

## 5. Conclusions

Due to the importance of breastfeeding’s role in maternal and infant health, it is no surprise that medical communities worldwide seek to encourage and prolong the length of time spent breastfeeding. The literature has also shown that though generally safe, maternal use of medications during lactation is one of the primary barriers to increased length of time breastfeeding [9]. This confirms a need for more training opportunities for medical providers on the safe use of medication in breastfeeding. Therefore, the InfantRisk Center and the American Heart Association set out to perform a training intervention to increase provider access to knowledge of safe medication practices, familiarity with the topic, and confidence in decision-making. This project minimally accomplished this goal and confirmed that a single education intervention is insufficient to make lasting impacts. A true longitudinal approach should be explored on this topic.

When surveyed, 100% of participants responded that this training was either important or very important to their respective practices. The study’s goal is confirmed by the overwhelmingly positive response to the intervention, as well as by the free text feedback that highlights the demand for similar training sessions. Specifically, the feedback emphasized the importance of incorporating information for infants within the hospital setting (neonates, premature infants, etc.). Data suggest the webinar interventions transiently increased confidence in medication use during lactation and confirmed the need for more training in this field. However, this information also confirms that simply providing a resource, like LactMed or the InfantRisk HCP app, is insufficient to support evidence-informed care for lactating patients. Further training on evidence-based medication safety resources for breastfeeding patients is critical for modern providers.

## Figures and Tables

**Table 1:** Results of survey questions of the analytic sample at baseline (pre-survey), one-week post-intervention (Post 1), and three months post-intervention.

Item	Pre-Survey N (%)	Post 1 N (%)	Post 2 N (%)	*p*-Value ^a^ (Pre-Post 1)	*p*-Value ^a^ (Pre-Post 2)
**Familiarity** for medication safety in those used for…					
Common conditions				n/a ^b^	0.797 (n = 11)
Not at all familiar (1)	2 (4.2)	n/a	1 (2.1)		
Slightly familiar (2)	11 (22.9)	n/a	6 (12.5)		
Very familiar (3)	19 (39.6)	n/a	7 (14.6)		
Extremely familiar (4)	7 (14.6)	n/a	4 (8.3)		
Missing	9 (18.8)	n/a	30 (62.5)		
Chronic conditions				n/a	1.000 (n = 18)
Not at all familiar (1)	3 (6.3)	n/a	0 (0.0)		
Slightly familiar (2)	16 (33.3)	n/a	7 (14.6)		
Very familiar (3)	21 (43.8)	n/a	9 (18.8)		
Extremely familiar (4)	6 (4.2)	n/a	2 (4.2)		
Missing	2 (4.2)	n/a	30 (62.5)		
Unique conditions				n/a	0.3008 (n = 18)
Not at all familiar (1)	29 (60.4)	n/a	10 (20.8)		
Slightly familiar (2)	15 (31.3)	n/a	5 (10.4)		
Very familiar (3)	1 (2.1)	n/a	2 (4.2)		
Extremely familiar (4)	1 (2.1)	n/a	1 (2.1)		
Missing	2 (4.2)	n/a	30 (62.5)		
Multiple simultaneous medications				n/a	0.680 (n = 18)
Not at all familiar (1)	15 (31.3)	n/a	6 (12.5)		
Slightly familiar (2)	27 (56.3)	n/a	9 (18.8)		
Very familiar (3)	3 (6.3)	n/a	2 (4.2)		
Extremely familiar (4)	1 (2.1)	n/a	1 (2.1)		
Missing	2 (4.2)	n/a	30 (62.5)		
**Confidence** to make recommendations in regard to medicine for…					
Common conditions				**0.000** (n = 39)	0.531 (n = 17)
Not at all confident (1)	4 (8.3)	0 (0.0)	3 (6.3)		
Slightly confident (2)	22 (45.8)	5 (10.4)	7 (14.6)		
Very confident (3)	16 (33.3)	27 (56.3)	6 (12.5)		
Extremely confident (4)	3 (6.3)	9 (18.8)	1 (2.1)		
Missing	3 (6.5)	7 (14.6)	31 (64.6)		
Chronic conditions				**0.000** (n = 36)	1.000 (n = 16)
Not at all confident (1)	6 (12.5)	1 (2.1)	2 (4.2)		
Slightly confident (2)	25 (52.1)	7 (14.6)	8 (16.7)		
Very confident (3)	10 (20.8)	27 (56.3)	6 (12.5)		
Extremely confident (4)	3 (6.3)	4 (8.3)	1 (2.1)		
Missing	4 (8.3)	9 (18.7)	31 (64.6)		
Unique conditions				**0.000** (n = 34)	0.656 (n = 16)
Not at all confident (1)	26 (54.2)	4 (8.3)	10 (20.8)		
Slightly confident (2)	13 (27.1)	19 (39.6)	5 (10.4)		
Very confident (3)	4 (8.3)	12 (25.0)	1 (2.1)		
Extremely confident (4)	0 (0.0)	3 (6.3)	1 (2.1)		
Missing	5 (10.4)	10 (20.8)	31 (64.6)		
Multiple simultaneous medications				**0.000** (n = 35)	0.688 (n = 16)
Not at all confident (1)	21 (43.8)	5 (10.4)	8 (16.7)		
Slightly confident (2)	19 (39.6)	13 (27.1)	6 (12.5)		
Very confident (3)	4 (8.3)	18 (37.5)	2 (4.2)		
Extremely confident (4)	0 (0.0)	2 (4.2)	1 (2.1)		
Missing	4 (8.3)	10 (20.8)	31 (64.6)		
When making recommendations, how often did you use the following **resources?**					
Personal experience				n/a	0.410 (n = 17)
Never (1)	16 (33.4)	n/a	8 (16.7)		
Seldom (2)	10 (20.8)	n/a	3 (6.3)		
Often (3)	15 (31.3)	n/a	4 (8.3)		
Always (4)	4 (8.3)	n/a	3 (6.3)		
Missing	3 (6.2)	n/a	30 (62.5)		
A trusted colleague				n/a	0.125 (n = 17)
Never (1)	9 (18.8)	n/a	4 (8.3)		
Seldom (2)	11 (22.9)	n/a	6 (12.5)		
Often (3)	19 (39.6)	n/a	6 (12.5)		
Always (4)	6 (12.5)	n/a	2 (4.2)		
Missing	3 (6.2)	n/a	30 (62.5)		
Internet search				n/a	0.699 (n = 17)
Never (1)	12 (25.0)	n/a	6 (12.5)		
Seldom (2)	17 (35.4)	n/a	3 (6.3)		
Often (3)	14 (29.2)	n/a	8 (16.7)		
Always (4)	2 (4.2)	n/a	1 (2.1)		
Missing	3 (6.2)	n/a	30 (62.5)		
Literature search				n/a	0.844 (n = 17)
Never (1)	7 (14.6)	n/a	5 (10.4)		
Seldom (2)	10 (20.8)	n/a	3 (6.3)		
Often (3)	20 (41.7)	n/a	6 (12.5)		
Always (4)	8 (16.7)	n/a	4 (8.3)		
Missing	3 (6.2)	n/a	30 (62.5)		
Information from drug manufacturer				n/a	0.281 (n = 18)
Never (1)	15 (31.3)	n/a	5 (10.4)		
Seldom (2)	15 (31.3)	n/a	8 (16.7)		
Often (3)	10 (20.8)	n/a	3 (6.3)		
Always (4)	6 (12.5)	n/a	2 (4.2)		
Missing	2 (4.1)	n/a	30 (62.5)		
Medical references				n/a	0.699 (n = 18)
Never (1)	15 (31.3)	n/a	6 (12.5)		
Seldom (2)	7 (14.6)	n/a	7 (14.6)		
Often (3)	17 (35.3)	n/a	3 (6.3)		
Always (4)	7 (14.6)	n/a	2 (4.2)		
Missing	2 (4.2)	n/a	30 (62.5)		
Lactation-specific reference				n/a	1.000 (n = 18)
Never (1)	4 (8.3)	n/a	1 (2.1)		
Seldom (2)	1 (2.1)	n/a	2 (4.2)		
Often (3)	13 (27.1)	n/a	4 (8.3)		
Always (4)	28 (58.3)	n/a	11 (22.9)		
Missing	2 (4.2)	n/a	30 (62.5)		

^a^ Wilcoxon signed-rank test for paired data, exact probability interpreted given n < 200; ^b^ Question not asked in Post-Survey 1 (see Supplementary Materials). The bolded values as *p* < 0.05.

**Table 2:** Baseline Characteristics.

Pre-Survey Characteristic	Baseline Sample n (%)	Analytic Sample n (%)
Role		
Nurse/Lactation Professional	63 (61.2%)	33 (68.8%)
Nurse Practitioner/Midwife	9 (8.7%)	3 (6.3%)
Physician	13 (12.6%)	3 (6.3%)
Other/Undisclosed	18 (17.5%)	4 (8.3%)
How frequently do you encounter the issue of medication use in breastfeeding women?		
Daily	18 (17.5%)	8 (16.7%)
Several times a week	23 (22.3%)	13 (27.1%)
Once a week	17 (16.5%)	8 (16.7%)
2–3 times a month	15 (14.6%)	9 (18.8%)
Monthly	9 (8.7%)	4 (8.3%)
Less often	15 (14.6%)	5 (10.4%)
Missing	6 (5.8%)	1 (2.1%)
To what extent do you have personal experience with breastfeeding, either for yourself, a family member, or a friend?		
A large amount (4)	54 (52.4%)	26 (54.2%)
A moderate amount	35 (34.0%)	17 (35.4%)
A small amount	12 (11.7%)	4 (8.3%)
No experience (1)	2 (1.9%)	1 (2.1%)
Missing	0 (0%)	0 (0%)

Differences between characteristic proportions for baseline and analytic samples were not statistically significant; chi-square *p* > 0.05.

## Data Availability

The data presented in this project are available on request from the corresponding author.

## Supplementary Materials

The following supporting information can be downloaded at https://www.mdpi.com/article/10.3390/ijerph20196850/s1, Table S1: Mixing Milk + Meds Quality Improvement Intervention Survey: language and matrix.
