# Supplementary material for: Mixing Meds and Milk: Evaluation of a Performance Gap Intervention for Provider Education in Breastfeeding and Maternal Medication Use
Source: Int J Environ Res Public Health. 2023 Sep 28;20(19):6850. doi: 10.3390/ijerph20196850 (PMC10572915; doi:10.3390/ijerph20196850)
Supplement: Supplementary file 1 [file ijerph-20-06850-s001.zip › ijerph-2585239-supplementary.pdf]

Table S1. Breastfeeding Training and App Survey Questions

The supplementary table presents the schedule of the survey items across different time points. In this table, each row represents a specific survey item or question, while columns denote distinct time points of surveys in relation to the training. An "X" denotes that the item was queried in the corresponding survey.

	2021			2022		
**Question**	**Pre-training survey**	**Post-training survey**	**3-month follow up survey**	**Pre-training survey**	**Post-training survey**	**3-month follow up survey**
To what extent do you have personal experience with breastfeeding, either for yourself, a family member, or a friend?<br><br>*A large amount of experience (4); A moderate amount of experience; A small amount of experience; No experience (1)*	X			X		
How frequently do you encounter the issue of medication use in breastfeeding women?<br><br>*Daily (6); Several times a week; Once a week; 2-3 times a month; Monthly; Less often (1); N/A*	X			X		
How **familiar** are you with evidence-supported medication safety for breastfeeding women in regard to:<br>• Common conditions (e.g., mastitis, depression, UTI, postpartum pain)<br>• Chronic conditions (e.g., hyptertension, diabetes, chronic pain)<br>• Unique conditions (e.g., transplant, STEMI)<br>• Multiple simultaneous medications	X		X	X		X

Extremely familiar (4); Very familiar; Slightly familiar; Not at all familiar (1); N/A						
How **<u>confident</u>** do you feel when making recommendations to breastfeeding women in regard to medications and:<br>• Common conditions (e.g., mastitis, depression, UTI, postpartum pain)<br>• Chronic conditions (e.g., hyptertension, diabetes, chronic pain)<br>• Unique conditions (e.g., transplant, STEMI)<br>• Multiple simultaneous medications<br><br>*Post-Webinar survey adjustment: [After the training, how confident do you feel about making future recommendations to breastfeeding women in regard to medications and:]<br><br>*Extremely confident (4); Very confident; Slightly confident; Not at all confident (1); N/A*	X	X*	X	X	X*	X
In the past 3 months, when addressing the issue of medication use in breastfeeding women, how often did you utilize the following resources?<br>• Personal experience<br>• A trusted colleague<br>• Internet search (e.g.,Google)<br>• literature search (e.g., PubMed, GoogleScholar)<br>• Information from drug manufacturer (e.g., package insert, drug representative)<br>• Medical reference (e.g., Epocrates, UpToDate, MedScape, Lexicomp)	X		X	X		X

• Lactation-specific reference (e.g., Lactmed, Hale's Medications and Mother's Milk, MommyMeds app, InfantRisk HCP app, Brigg's Drugs in Pregnancy in Lactation)<br>• Other: ____________<br><br>*Always (4); Often; Seldom; Never(1)*						
Please rate the extent to which the presenter was knowledgeable, organized, and effective in his presentation.<br><br>*Very good; Good; Fair; Poor*		X			X	
To what extent were the educational objectives achieved?<br><br>*Very good; Good; Fair; Poor*		X			X	
How likely will the information presented be integrated into your practice?<br><br>*Very likely (4); Somewhat likely; Somewhat unlikely; Very unlikely (1)*		X			X	
What did you like or dislike about the InfantRisk app? (free text)			X			X
How helpful did you find the following components of this program?<br>• Webinar training<br>• InfantRisk HCP app<br><br>*Very helpful (3); somewhat helpful; Not helpful (1); Not applicable*			X			
How important do you think it is for healthcare providers to have training on medications and breastfeeding?			X			X

Very important (4); Important; Unimportant; Very unimportant (1)						
Do you feel you need additional training in the medical management of breastfeeding women? (yes/no)<br>If yes: What types of training would you find most useful? (free text)			X			X
Is there anything else you would like to tell us? [free text]	X	X	X	X	X	X
Which field most closely aligns with your current work?				X	X	X
Have you used the InfantRisk HCP app since the webinar?			X			X
Role					X	X
ParticipantID	X	X	X	X	X	X
